# Can soda ash dumping grounds provide replacement habitats for digger wasps (Hymenoptera, Apoidea, Spheciformes)?

**DOI:** 10.1371/journal.pone.0175664

**Published:** 2017-04-19

**Authors:** Lucyna Twerd, Maciej Krzyżyński, Barbara Waldon-Rudzionek, Piotr Olszewski

**Affiliations:** 1 Department of Ecology, Institute of Environmental Biology, Kazimierz Wielki University, Bydgoszcz, Poland; 2 Chair of Ecology and Biogeography, Faculty of Biology and Environmental Protection, Nicolaus Copernicus University in Toruń, Toruń, Poland; 3 Department of Botany, Institute of Environmental Biology, Kazimierz Wielki University, Bydgoszcz, Poland; University of Roehampton, UNITED KINGDOM

## Abstract

**Background:**

Published sources document a loss of biodiversity at an extreme rate, mainly because natural and semi-natural ecosystems are becoming fragmented and isolated, thus losing their biological functions. These changes significantly influence biological diversity, which is a complex phenomenon that changes over time. Contemporary ecologists must therefore draw attention to anthropogenic replacement habitats and increase their conservation status. In our studies we show the positive role of soda ash dumping grounds as an alternative habitat for digger wasps, especially the thermophilic species.

**Methodology/Principal findings:**

In the years 2007–2010 we carried out investigations in postindustrial soda ash dumping grounds located in Central Poland. We demonstrated that these areas serve as replacement habitats for thermophilic species of *Spheciformes* and, indirectly, for their potential prey. The studies were conducted in three microhabitat types, varying in soil moisture, salinity and alkalinity, that were changing in the course of ecological succession. We trapped 2571 specimens belonging to 64 species of digger wasps. Species typical of open sunny spaces comprised 73% of the whole inventory. The obtained results suggest that the stage of succession determines the richness, abundance and diversity of *Spheciformes*. The most favorable conditions for digger wasps were observed in habitats at late successional stages.

**Conclusions/Significance:**

Our results clearly showed that these habitats were replacement habitats for thermophilous Spheciformes, including rare taxa that require genetic, species and ecosystem protection, according to the Biodiversity Convention. We showed that some types of industry might play a positive role in the preservation of taxa in the landscape, and that even degraded industrial wasteland can replace habitats under anthropopressure, serving as refugia of biological diversity, especially for disturbance-dependent species.

## Introduction

The development of agricultural systems, ongoing urbanization, industrialization and globalisation over the past century have led to an increase in the area of degraded lands, the fragmentation of natural vegetation and the loss of natural and semi-natural habitats [1]. Ash dumps, stone, gravel and sand quarries, together with urban and agricultural areas, are the most typical anthropogenic habitats that provide refuges for biological diversity [2–5]. Their role as replacement habitats to preserve insects biodiversity has recently attracted worldwide attention [6–12].

Postindustrial soda ash dumping sites, formed as a result of industrial processes for the production of sodium carbonate, calcium chloride, precipitated calcium carbonate and calcium fertilizers [13], represent a very special type of anthropogenic habitat.

The waste silt that remains after producing soda ash contains about 80% calcium carbonate (CaCO_3_) and is highly alkaline. The concentration of salts in these silts depends on the age of the deposit. The alkaline and salty waste from the soda production process is also rich in compounds such as CaSO_4_, Ca(OH)_2_, Fe(OH)_3_, silicates and aluminosilicates, and the supernatant is a solution of NaCl, KCl, NH_4_OH, Na_2_SO_4_, NaOH or MgCl_2_ [14]. In our study these sediments had been stored and drained next to the factories in sedimentation basins, which are in the form of slag heaps 16 m high, covering ca. 100 ha in Inowrocław and ca. 125 ha in Janikowo. The sediments are among the most environmentally harmful waste and they do not easily develop a vegetation cover [15]. The waste harms the surrounding environment mostly by permeating soda and chlorine salts into the substrate. Dust blown from the sediment is also hazardous [16]. As salinity and alkalinity decrease over time, this unfavourable biotope is slowly colonised by a typical synanthropic vegetation [15]. Since it might take decades to revegetate the dumps, technical and biological recultivation is often recommended [16–17].

The role of soda ash dump sites as replacement habitats for animal species is not well understood yet. A few pilot studies have suggested that such sites represent suitable areas for Aculeate diversity [18, 19]. In our first work we studied the diversity of Chrysididae in areas of the soda and calcareous industry, while in the second, we conducted some preliminary research on Aculeata biodiversity in areas of the soda industry, where we found only 38 species of Crabronidae. These studies were conducted without an evaluation of the role of microhabitats and phytoindicatory methods.

Digger wasps, which are the subject of our study, occur in various habitats, concentrating in places rich in prey for the larval stages and abundant in nectar sources for adult provisions. Digger wasps, like other Aculeata often rely on multiple habitats, feeding in one but gathering building materials in another [20]. The complexity and diversity requirements of life make Sphecidae a particularly vulnerable group to changes in the environment [21, 22]. The vast majority of species of digger wasps prefer open areas, including dry and warm grasslands, although some of them are associated with forests [23]. Two hundred and thirty-three species of *Spheciformes* have been reported in Poland [24–30].

The paper by Wiśniowski [30] offers the most comprehensive resource for distribution and diversity of digger wasps in Poland. In addition, there is also literature available on the impact of environmental factors on the fauna and flora present in the areas under industrial anthropopressure in Poland and Europe [3, 5, 7, 18, 31, 32], but there is no information on the Spheciformes living on soda ash dumping sites.

Habitats provided by soda waste dumps are unusual. The high content of calcium carbonate in the soil changes its thermal properties, supporting the development of thermophilic habitats and promotes thermophilic fauna, but excessive moisture in the ground can reduce the number of nesting places available for many Spheciformes species. Therefore, it is very important to understand the mechanisms influencing the diversity of the selected species groups in the studied areas.

In this paper, we will attempt to answer the following questions: (1) what are the habitat, nesting and food preferences of the *Spheciformes* occurring in the areas under the influence of the soda ash industry? (2) How does anthropogenic pressure, measured in terms of soil degradation and successional stages on soda ash dumping sites (basins), determine the richness, and abundance of digger wasps? (3) What microhabitat structure provides the optimum nesting conditions for *Spheciformes* living on soda ash dumping sites?

We formulated a hypothesis that soda ash dumps, due to their favorable microclimate, can act as replacement habitats for thermophilic digger wasps. Our other hypothesis assumed that microhabitats that are excessively moist, salty and alkaline are not preferred by digger wasps although these sites can be favored by their potential prey [33].

On the other hand, stress factors negatively affect some flora and fauna species, thus, it is very important to understand the habitat preferences of the species as well as their adaptive potential in an anthropogenic environment.

## Materials and methods

All necessary permits were obtained for the described study, which complied with all relevant regulations. Permits L. Twerd 297/2007/2008 and L. Twerd 356/2009/2010 for conducting research on the area of the Soda Polska Ciech company in Inowrocław and Janikowo.

### Study area

**Fig 1:**
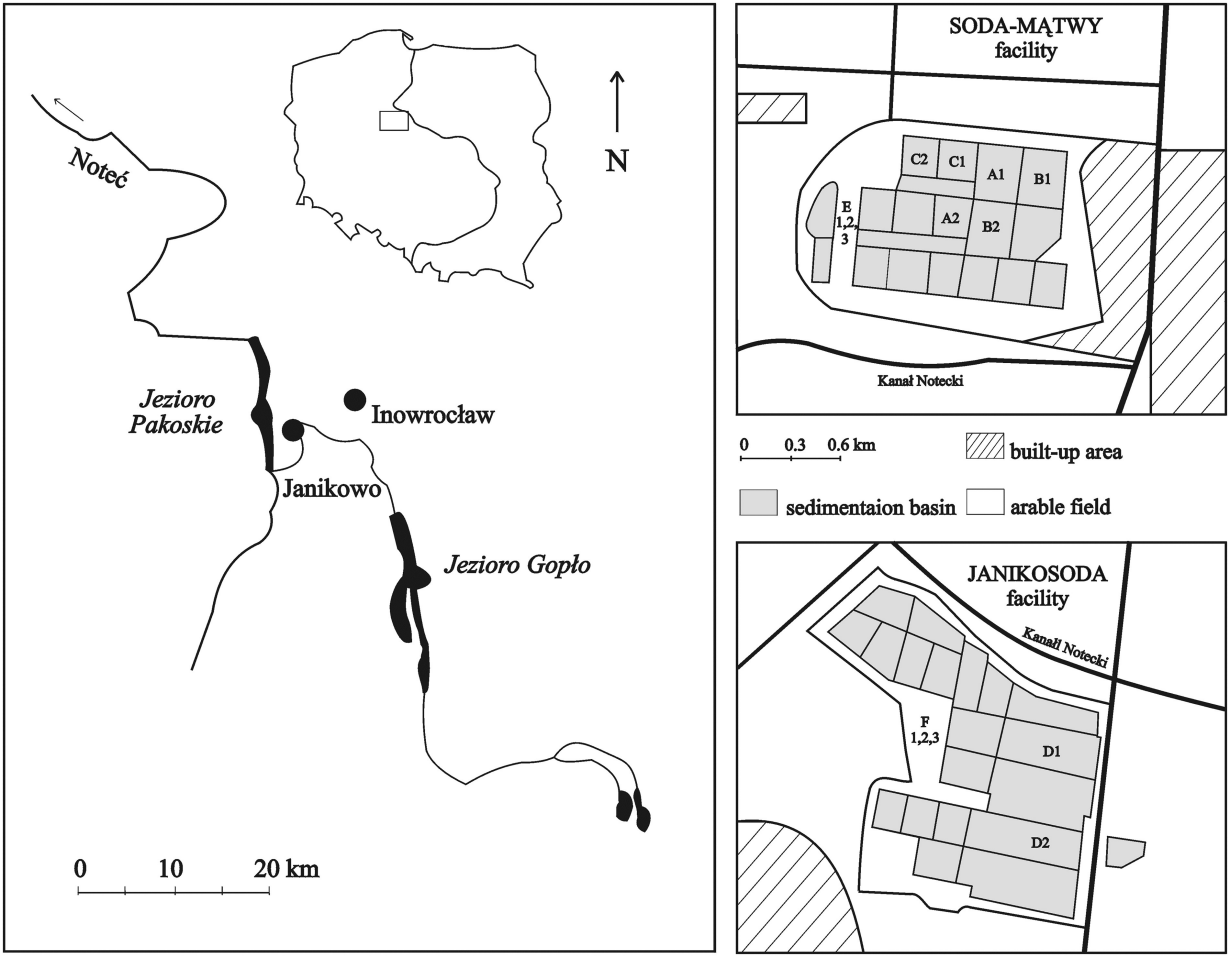
The study area with soda ash wastelands in the Kujawy region of central Poland. Study sites: **microhabitat 1**, sites A, B: succession stage I (A1, B1–2009 year, A2, B2–2010 year); m**icrohabitat 2**, sites C, D: succession stage II (C1, D1–2009 year, C2, D2–2010 year); **microhabitat 3**, sites E, F: succession stage III (E1, F1–2007 year, E2, F2–2008 year, E3, F3–2009 year) (see Tables 1 and 2).

The studies were carried out in the Kujawy region of Central Poland (Fig 1), in the area of the Mid-Polish Anticlinorium (Kujawy-Pomeranian Zone), rich in salt plugs, Jurassic lime and marl sediments that are used by the soda ash industry. The investigations were conducted in soda ash wastelands that were still in use and unique in Poland, owned by Soda Polska CIECH sp. z o.o., with two soda ash facilities: SODA MĄTWY in Inowrocław (N: 52° 45’ 17.03”, E: 18° 14’ 16”) and JANIKOSODA in Janikowo (N: 52° 46’ 27.61”, E: 18° 7’ 21.63”).

Highly saline, alkaline and moist solid waste and industrial wastewater are disposed of and hydraulically drained into sedimentation basins in areas next to the factory sites. The waste heaps are up to 16 m high. In total, the dump sites cover ca. 100 ha in Inowrocław and ca. 125 ha in Janikowo.

Our studies covered two soda ash dumping sites (in Inowrocław and Janikowo). The insects were collected in three microhabitats undergoing spontaneous succession: **microhabitats 1:** succession stage I [A, B] soda ash waste, herbaceous vegetation coverage < 60%; **microhabitats 2:** succession stage II [C, D–the slopes of heaps] soda ash waste, herbaceous vegetation coverage > 60%; **microhabitats 3:** succession stage III [E, F] soda ash waste, herbaceous vegetation coverage > 80%, tree coverage > 5% (Fig 1, Tables 1 and 2).

**Table 1:** Characteristics of the three microhabitats and values of the component “anthropopressure” extracted by PCA: [1]—Soil degradation, [2]—Succession stage.

**Microhabitat**	**Site**	**r = 200 m** **Percentage of plant species that are indicators of**			**[1]** **Component** **"soil degradation"**
		salty soils	alkaline soils	moist soils	
1	A	17.24	31.03	13.79	0.609
	B	15.00	33.12	12.02	0.568
2	C	5.26	19.04	10.52	0.237
	D	2.56	19.95	9.52	0.065
3	E	0.00	14.78	7.69	-0.351
	F	0.00	12.82	7.30	-0.363
**Microhabitat**	**Site**	% of bare surface with no plants	% of herbaceous vegetation	% of woodlands	**[2]** **Component** **"succession stage"**
1	A	50	50	0	-0.495
	B	45	55	0	-0.472
2	C	30	70	0	-0.388
	D	20	80	0	-0.308
3	E	5	85	10	0.531
	F	5	90	5	0.383

**Table 2:** The list of Spheciformes species in the areas influenced by the soda ash industry. The ecological classification of the species was based on the works of Jacobs and Oehlke [39], Schmid-Egger et al. [40] and Blösch [23]: 1–18 (eurytopic open areas species), 19–47 (stenotopic open areas species), 48–64 (stenotopic forest species). Threat categories according to Fauna of Poland [46]: EX?–probably extinct; VU–vulnerable, NT–nearly threatened; DD–deficient data; vrm–very rare, rm–rare.Nesting preferences: en–endogeic species (nesting in the ground), (en)–cleptoparasite endogeic species, hy–hypergeic species (nesting above ground). Food preferences: Ara–Araneae, Dip–Diptera, Col–Coleoptera, He-Ap–He-an–Hemiptera-another, Hym–Hymenoptera, cl–cleptoparasite, Ort–Orthoptera, Ort-Bla–Orthoptera & Blattodea, Lep–Lepidoptera.

No	Species	Status	Nesting	Food	Inowrocław				Janikowo	
					SODA-MĄTWY facility				JANIKOSODA facility	
					A	B	C	E	D	F
					2009–2010	2009–2010	2009–2010	2007–2009	2009–2010	2007–2009
1	*Alysson spinosus* (Panzer, 1801)	-	en	He-an	-	-	-	1	-	-
2	*Ammophila campestris* Latreille, 1809	-	en	Hym	-	-	-	2	-	-
3	*Ammophila sabulosa* (Linnaeus, 1758)	-	en	Lep	-	1	5	5	19	6
4	*Argogorytes mystaceus* (Linnaeus, 1761)	-	en	He-an	1	1	-	6	-	-
5	*Astata boops* (Schrank, 1781)	-	en	He-an	9	2	14	15	4	-
6	*Cerceris quinquefasciata* (Rossi, 1792)	-	en	Col	4	26	25	31	58	16
7	*Cerceris rybyensis* (Linnaeus, 1771)	-	en	Hym	22	30	175	171	42	46
8	*Crabro cribrarius* (Linnaeus, 1758)	-	en	Dip	139	15	175	34	7	-
9	*Diodontus minutus* (Fabricius, 1793)	-	en	He-Ap	-	-	1	1	3	3
10	*Diodontus tristis* (Curtis, 1829)	-	en	He-Ap	-	-	-	1	-	4
11	*Harpactus lunatus* (Dahlbom, 1832)	-	en	He-an	-	-	-	-	3	-
12	*Lindenius albilabris* (Fabricius, 1793)	-	en	He-an	-	1	4	13	8	10
13	*Lindenius pygmaeus* (Rossi, 1794)	-	en	Hym	-	-	-	45	4	1
14	*Mellinus crabroneus* (Thunberg, 1791)	-	en	Dip	1	-	-	-	-	2
15	*Oxybelus bipunctatus* Olivier, 1812	-	en	Dip	-	-	1	25	-	-
16	*Oxybelus trispinosus* (Fabricius, 1787)	-	en	Dip	-	-	-	1	-	-
17	*Philanthus triangulum* (Fabricius, 1775)	-	en	Hym	6	1	7	9	3	5
18	*Tachysphex pompiliformis* (Panzer, 1805)	-	en	Ort	-	-	5	9	13	12
19	*Cerceris arenaria* (Linnaeus, 1758)	-	en	Col	-	1	-	1	-	1
20	*Cerceris flavilabris* (Fabricius, 1793)	vrm NT NT	en	Col	-	-	1	-	-	-
21	*Cerceris interrupta* (Panzer, 1799)	-	en	Col	-	-	-	-	2	-
22	*Crabro peltarius* (Schreber, 1784)	-	en	Dip	-	-	-	4	1	1
23	*Crabro scutellatus* (Scheven, 1781)	-	en	Dip	-	-	1	27	4	1
24	*Diodontus luperus* Shuckard, 1837	-	en	He-an	-	-	-	1	-	1
25	*Dryudella stigma* (Fabricius, 1793)	-	en	He-an	1	-	3	10	-	-
26	*Entomognathus brevis* (van der Linden, 1829)	-	en	Col	-	-	-	2	1	1
27	*Gorytes quinquefasciatus* (Panzer, 1798)	-	en	He-an	-	-	1	-	-	2
28	*Harpactus laevis* (Latreille, 1792)	vrm VU	en	He-an	-	-	-	-	1	-
29	*Lestica alata* (Panzer, 1797)	VU	en	Lep	-	2	4	9	1	4
30	*Lindenius panzeri* (Van der Linden, 1829)	-	en	Dip	-	-	2	2	-	1
31	*Mimesa bicolor* (Jurine, 1807)	-	en	He-an	1	-	1	-	-	-
32	*Nysson dimidiatus* Jurine, 1807	-	(en)	Cl	-	-	-	-	-	1
33	*Nysson interruptus (Fabricius*, *1798)*	-	(en)	Cl	2	-	2	12	1	-
34	*Nysson maculosus* (Gmelin, 1790)	-	(en)	Cl	12	3	66	81	14	13
35	*Nysson niger* Chevrier, 1868	NT	(en)	Cl	-	-	2	7	-	1
36	*Nysson tridens* Gerstaecker, 1867	-	(en)	Cl	-	-	-	1	-	-
37	*Oxybelus mandibularis* Dahlbom, 1845	-	en	Dip	-	-	-	60	2	1
38	*Oxybelus quatuordecimnotatus* Jurine, 1807	-	en	Dip	5	3	16	354	9	21
39	*Oxybelus uniglumis* (Linnaeus, 1758)	-	en	Dip	-	-	-	3	5	1
40	*Oxybelus variegatus* Wesmael, 1852	VU	en	Dip	-	-	-	3	1	1
41	*Podalonia affinis* (Kirby, 1798)	-	en	Lep	1	1	5	5	9	2
42	*Podalonia hirsuta* (Scopoli, 1763)	-	en	Lep	1	-	-	-	1	-
43	*Tachysphex fulvitarsis* (Costa, 1867)	EX?	en	Ort	-	-	-	2	1	1
44	*Tachysphex nitidus* (Spinola, 1805)	-	en	Ort-Bla	-	-	-	-	-	2
45	*Tachysphex psammobius* (Kohl, 1880)	NT	en	Ort	-	1	-	1	-	-
46	*Tachysphex unicolor* (Panzer, 1809)	vrm	en	Ort	-	-	-	1	-	-
47	*Astata minor* Kohl, 1885	-	en	He-an	-	-	3	1	-	-
48	*Crossocerus cetratus* (Shuckard, 1837)	-	hy	Dip	-	-	-	-	1	-
49	*Ectemnius confinis* (Thomson, 1870)	vrm	hy	Dip	-	-	-	17	-	6
50	*Ectemnius continuus* (Fabricius, 1804)	-	hy	Dip	-	-	-	2	20	5
51	*Ectemnius dives* (Lepeletier & Brullé, 1834)	-	hy	Dip	1	-	-	2	-	1
52	*Ectemnius rubicola* (Dufour & Perris,1840)	-	hy	Dip	-	-	-	2	1	-
53	*Nysson trimaculatus* (Rossi, 1790)	-	(en)	Cl	-	-	-	1	1	-
54	*Passaloecus clypealis Faester*, 1947	DD	hy	He-Ap	-	-	-	3	-	-
55	*Passaloecus singularis* Dahlbom, 1844	-	hy	He-Ap	-	-	-	3	-	1
56	*Pemphredon austriaca* (Kohl, 1888)	vrm	hy	He-Ap	1	-	2	5	4	3
57	*Pemphredon inornata* Say, 1824	-	hy	He-Ap	-	-	-	-	1	-
58	*Pemphredon lethifer* (Shuckard, 1837)	-	hy	He-Ap	-	-	-	102	3	32
59	*Pemphredon morio* Van der Linden, 1829	-	hy	He-Ap	-	-	-	1	1	3
60	*Solierella compedita* (Piccioli, 1869)	vrm	hy	He-an	-	-	-	2	3	-
61	*Trypoxylon attenuatum* Smith, 1851	-	hy	Ara	-	-	-	1	-	-
62	*Trypoxylon deceptorium* Antropov, 1991	-	hy	Ara	-	-	-	5	-	3
63	*Trypoxylon figulus* (Linnaeus, 1758)	rm	hy	Ara	-	-	-	20	37	3
64	*Trypoxylon minus* (De Beaumont, 1945)	-	hy	Ara	1	-	1	48	40	26
**Species richness**					**17**	**14**	**25**	**52**	**37**	**38**
**Abudance**					**208**	**88**	**524**	**1176**	**329**	**246**
**Abudance/year**					**104.0**	**44.00**	**262.00**	**392.00**	**164.50**	**82.00**
**The estimated number of species Chao1 (in respect of abudance)**					**57.50**	**26.25**	**37.25**	**62.57**	**79.25**	**66.13**

Particular microhabitats differed also in the degree of soil moisture, salinity and alkalinity. Physical and chemical parameters of the soil were determined with the use of Ellenberg’s indicator values [34] (*see* Methods *- Habitat factors—Habitat conditions*).

### Habitat factors

In each microhabitat, the selected environmental parameters were analyzed within a 200 m radius around the traps. This method is regarded as the most suitable one for the analysis of environmental conditions in biotopes of limited diversity [35].

The following parameters were analyzed:

1. *Habitat structure*, i.e. the percent coverage of: herbaceous vegetation, dicotyledonous herbaceous vegetation (> 25 cm, < 25 cm high), grass vegetation (>50 cm, < 50 cm high), trees (> 2.5 m, < 2.5 m) and bare land.
2. *Habitat conditions*, determined with the use of Ellenberg’s indicator values [34]. The values for vascular plants were taken from Zarzycki et al. [36]. Three environmental factors, soil salinity **(S)**, soil acidity **(R)**, and soil humidity **(F)**, were described by scale of indicator values [36] (See S1 Appendix)–**(S)** soil salinity (the content of NaCl in the soil): S1 (species tolerant of elevated NaCl content), S2 (species associated with saline soils); **(R)** soil acidity: R2 (acidic soils, 4 ≤ pH < 5), R3 (moderately acid, 5 ≤ pH < 6), R4 (neutral, 6 ≤ pH ≤ 7), R5 (alkaline, pH > 7); **(F)** soil humidity: F2 (dry), F3 (fresh), F4 (moist), F5 (wet) [36]. Species were counted in individual ranges, and the calculation takes into account only the ranges that are most numerous in species.

The calculations were based on floristic inventories conducted within the area of the 200 m radius (see S1 Appendix for the species list).

### Insect catchers

The insects were collected with the use of Moericke traps (white plastic pans, 20 cm in diameter) filled to ¾ with a mixture of water (94.2%), ethylene glycol (5.6%) and a detergent (0.2%). The use of coloured pans is one of the most common methods of monitoring Aculeates. Yellow and white pans are the most effective ones for collecting Aculeata [37–38].

Twelve traps were placed at each microhabitat every ten metres along a transect line. Each trap was mounted on a metal rod, 60 cm above ground, at the level of the herbaceous vegetation. Insects were removed from the traps every 10 days, from April to September, in the years 2007–2010 (Table 2). The total number of samples was 1728.

**Fig 2:**
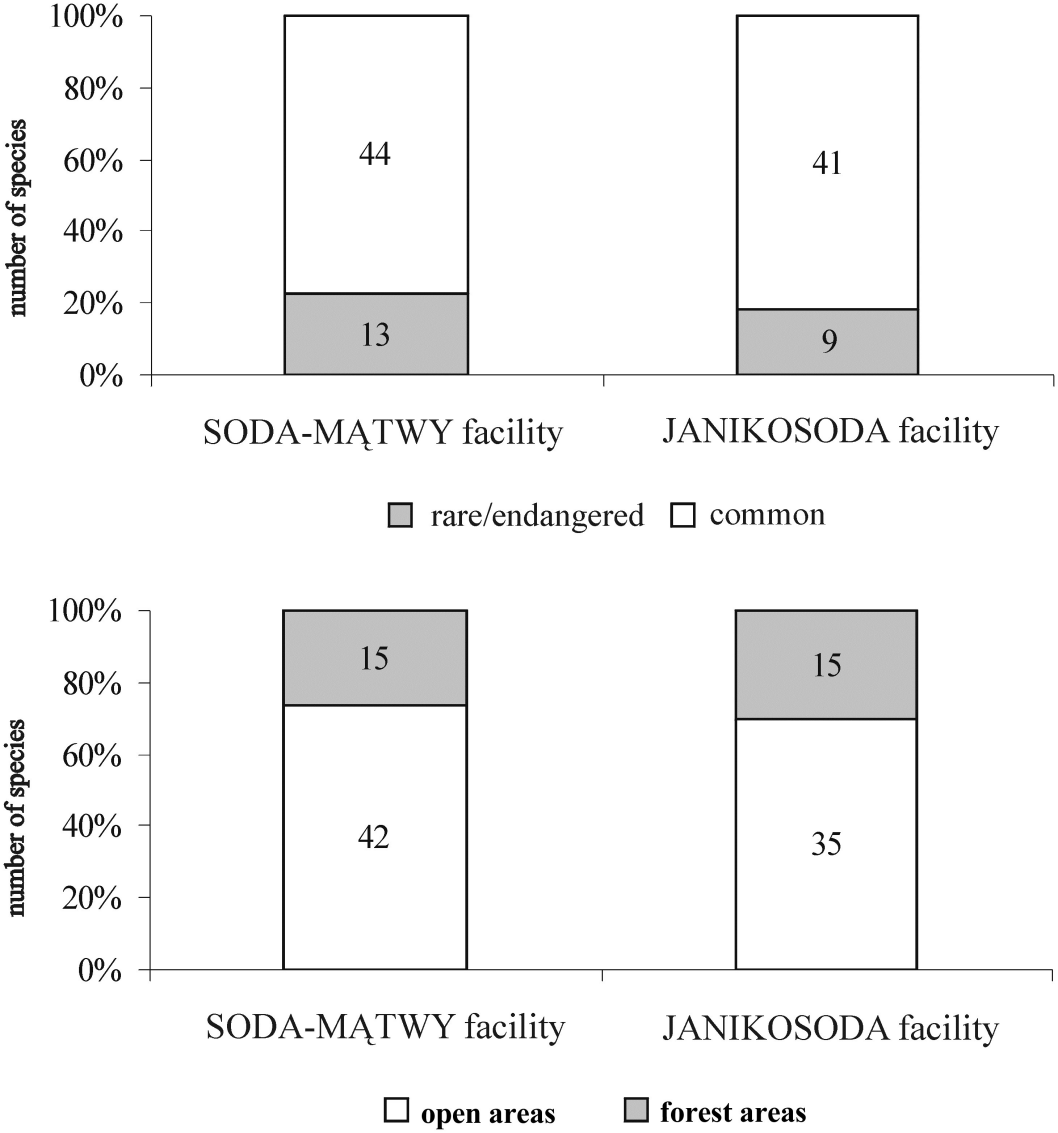
The percentage of *Spheciformes* in the areas affected by the soda ash industry. (See Table 2).

In our studies, we assumed that the selected microhabitat types are preferred by different species because they offer different nesting conditions for Spheciformes. The ecological classification of the species was based on the works of Jacobs and Oehlke [39], Schmid-Egger *et al*. [40] and Blösch [23] (Fig 2).

We also compared the larvae food spectrum of species we found on the soda ash wastelands to the data occuring whole polish fauna of digger wasps [23, 41].

### Statistical analysis

The species richness was assessed using rarefaction curves [42]. The computations were performed with EstimateS software [43]. The extrapolated species richness (the number of observed species and the unobserved ones) was estimated using a Chao1 estimator [44].

Numerical analysis of the data was carried out with the use of CANOCO v. 4 software [45]. Before the analysis, the dependent variable (species) was replaced by log(x). To reveal the gradient of total variation in the data set, the indirect DCA analysis was performed. The gradient length in the data was 2.63 SD. For gradients < 3 SD it is recommended to use linear methods [45]. Direct ordination was performed with the use of the Redundancy Analysis method (RDA). The significance of variables and canonical axes were examined using the Monte Carlo Permutation Test with 1000 repetitions.

**Fig 3:**
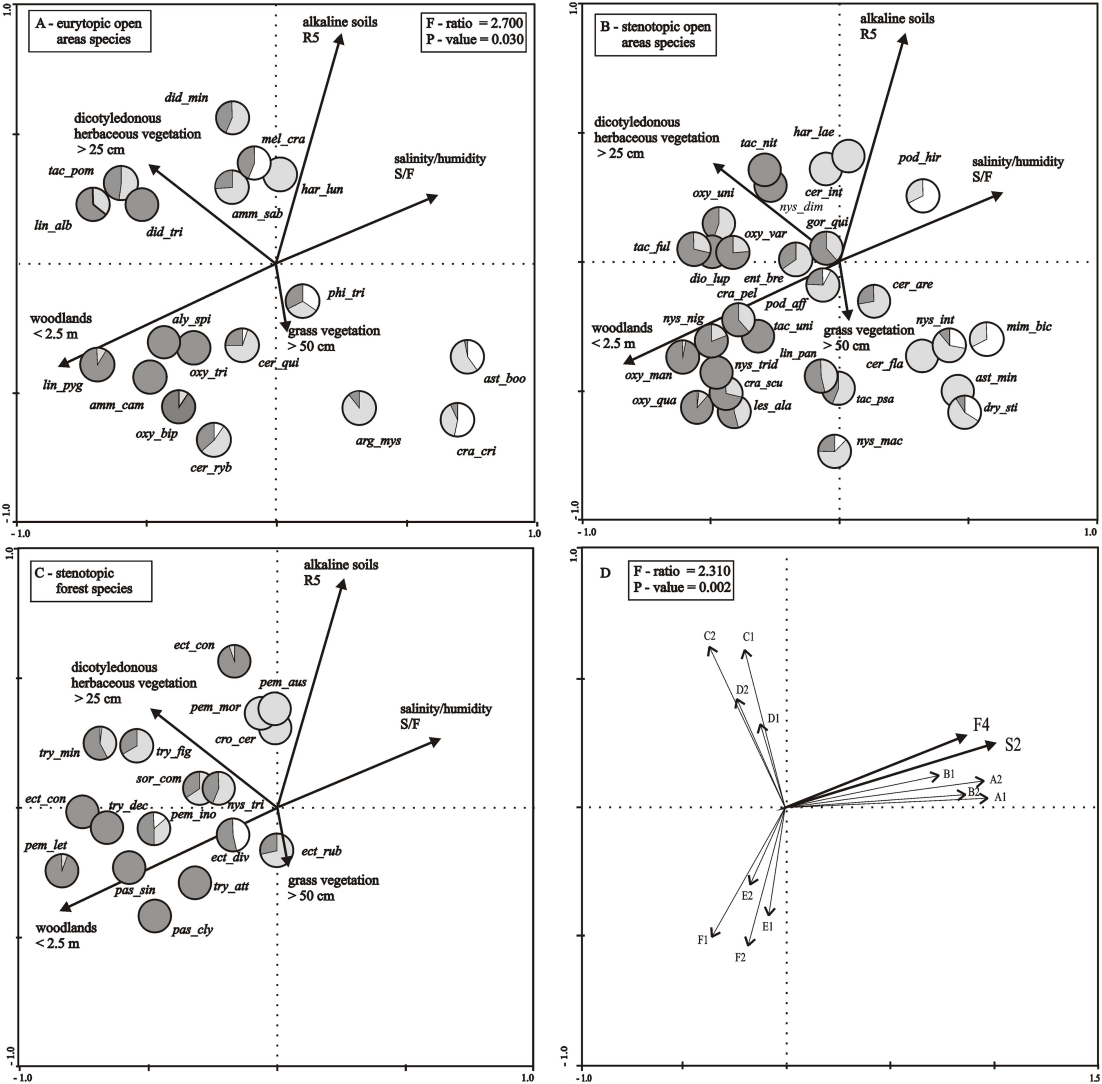
a-d. RDA ordination diagram showing the loadings of *Spheciformes* species on the axes I and II and the vectors representing the analyzed habitat parameters. **Pie charts show the percentage of these species in microhabitat 1 (white), microhabitat 2 (gray) and microhabitat 3 (black). The variables shown in the figure explained 66.8% of the variation in *Spheciformes* species data (the sum of all canonical eigenvalues: 0.668, the sum of all eigenvalues: 1.000; 0.668/1.000 = 0.668 i. e. 66.8%)**. Microhabitat 1: succession stage I soda ash waste, herbaceous vegetation coverage < 60%; microhabitat 2: succession stage II soda ash waste, herbaceous vegetation coverage > 60%; microhabitat 3: succession stage III soda ash waste, herbaceous vegetation coverage > 80%, tree coverage > 5%. (R) soil acidity: R5 (alkaline, pH > 7). S/F: salinity/moisture (*See* Methods). Species name abbreviations: aly_spi—*Alysson spinosus*; amm_cam—*Ammophila campestris*; amm_sab—*Ammophila sabulosa*; arg_mys—*Argogorytes mystaceus*; ast_boo—*Astata boops*; ast_min—*Astata minor*; cer_are—*Cerceris arenaria*; cer_fla—*Cerceris flavilabris*; cer_int—*Cerceris interrupta*; cer_ryb—*Cerceris rybyensis*; cer_qui—*Cerceris quinquefasciata*; cra_cri—*Crabro cribrarius*; cra_pel—*Crabro peltarius*; cra_scu—*Crabro scutellatus*; cro_cer—*Crossocerus cetratus*; dio_lup—*Diodontus luperus*; dio_min—*Diodontus minutus*; dio_tri -*Diodontus tristis*; dry_sti—*Dryudella stigma*; ect_conf—*Ectemnius confinis*; ect_con—*Ectemnius continuus*; ect_div—*Ectemnius dives*; ect_rub—*Ectemnius rubicola*; ent_bre—*Entomognathus brevis*; gor_qui—*Gorytes quinquefasciatus*; har_lae—*Harpactus laevis*; har_lun—*Harpactus lunatus*; les_ala—*Lestica alata*; lin_alb—*Lindenius albilabris*; lin_pan—*Lindenius panzeri*; lin_pyg—*Lindenius pygmaeus*; mel_cra—*Mellinus crabroneus*; mim_bic—*Mimesa bicolor*; nys_dim—*Nysson dimidiatus*; nys_int—*Nysson interruptus*; nys_mac—*Nysson maculosus*; nys_nig—*Nysson niger*; nys_trid*—Nysson tridens*; nys_tri—*Nysson trimaculatus*; oxy_bip—*Oxybelus bipunctatus*; oxy_man—*Oxybelus mandibularis*; oxy_tri—*Oxybelus trispinosus*; oxy_qua—*Oxybelus quatuordecimnotatus*; oxy_uni—*Oxybelus uniglumis*; oxy_var—*Oxybelus variegatus*; pas_cly—*Passaloecus clypealis*; pas_sin—*Passaloecus singularis*; pem_aus—*Pemphredon austriaca*; pem_ino—*Pemphredon inornata*; pem_let—*Pemphredon lethifer*; pem_mor—*Pemphredon morio*; phi_tri—*Philanthus triangulum*; pod_aff—*Podalonia affinis*; pod_hir—*Podalonia hirsuta*; sor_com*—Solierella compedita*; tac_ful—*Tachysphex fulvitarsis*; tac_nit—*Tachysphex nitidus*; tac_pom—*Tachysphex pompiliformis*; tac_psa—*Tachysphex psammobius*; tac_uni—*Tachysphex unicolor*; try_att*—Trypoxylon attenuatum*; try_dec—*Trypoxylon deceptorium*; try_fig—*Trypoxylon figulus*; try_min—*Trypoxylon minus*.Principal Components Analysis (PCA): microhabitat 1: (A1, A2, B1, B2), microhabitat 2: (C1, C2, D1, D2), microhabitat 3: (E1, E2, F1, F2).

RDA analysis was conducted for parameters describing the analyzed microhabitats: 8 variables describing the *Habitat structure* (*see* Methods *–Habitat factors*), and 8 parameters reflecting the *Habitat conditions S1; S2; R3; R4; R5; F2; F3; F4* (*see* Methods *–Habitat factors*). *Habitat conditions* R2 and F5 were ignored due to the least number (statistically insignificant) of indicator species of digger wasps observed. Due to the fact that variables S2 and F4 were colinear, they were combined into one variable S/F (salinity/moisture index) using the principal component analysis (PCA). The correlation between variables S2 and F4 is shown on the PCA plot, (Fig 3d).

The next step in the analysis was determining two environmental gradients that reflect the degree of anthropopressure. To this end, the selected and correlated variables indicating the anthropopressure level (percentage of bare land with no plants, herbaceous vegetation, woodlands) and (S1, S2, R5, F4) (*see* Methods *–Habitat factors–Habitat conditions*) were combined with the use of principal component analysis into two components referred to as “soil degradation” and “succession stage” (Table 2). These two secondary variables explained 86% of the total variance of the original variables. Positive values of the component “soil degradation” indicate very salty, alkaline and moist microhabitats, whereas negative values correspond to microhabitats that are not salty and are dry (Table 1). Negative values of the component “succession stage” indicate succession stages I and II, while positive values correspond to the third stage of the succession.

A general linear model (GLM) was used to visualize the changes in species richness, abundance and diversity along the determined environmental gradients.

## Results

In total, 2571 specimens of two families, *Sphecidae* and *Crabronidae*, from 64 species (28% of the domestic Polish fauna) were trapped (Table 2). The estimated number of species (Chao1 in respect of abundance) is presented in Table 2.

In agreement with our hypothesis, most of the *Spheciformes* found are thermophilic species associated with open spaces. These taxa accounted for as much as 73.43% of the whole inventory, including both stenotopic and eurytopic species associated with open spaces (45.31% and 28.12%, respectively) (Fig 2).

### Habitat preferences

Three factors significantly affected the habitat preferences of the species (Table 3). In habitats of low soil salinity and alkalinity we observed every digger wasp species found in our studies, regardless of their ecological status. Their preferences focused mainly on the habitat (vegetation) structure (Fig 3a, 3b and 3c).

**Table 3:** Results of stepwise selection of variables and a Monte Carlo Permutation Test–Analysis of the significance of the effect of the studied variables on the occurrence of different species of *Spheciformes*; marked variables were significant at p < 0.05.

Variables	RDA		
	Level of significance	Variation	% of explained variation
woodlands < 2.5 m	0.001	0.25	3.01
R5 –alkaline soils (6≤pH<7)	0.018	0.16	2.14
S/F–salinity/humidity	0.037	0.12	1.81
dicotyledonous vegetation > 25 cm	0.110	0.10	1.56
grass > 50 cm	0.743	0.04	0.62

For example, eurytopic species associated with open areas, such as *L*. *pygmaeus*,. *campestris*, *A*. *spinosus*, *O*. *trispinosus* or *O*. *bipunctatus*, dominated in microhabitats with a low coverage of low arborescent vegetation (< 2.5 m) (Fig 3a). These taxa along with *D*. *tristis*, *L*. *albilabris* and *T*. *pompiliformis*, dominated in microhabitats of the succession stage III, the latter three species being associated mainly with flowering dicotyledonous plants (vegetation layer height > 25 cm). Only a few species, *P*. *triangulum*, *A*. *mystaceus*, *C*. *cribrarius* and *A*. *boops*, were found in the habitats with grass vegetation (layer height > 50 cm) (Fig 3a).

The analysis of the distribution of stenotopic species associated with open areas or forests revealed relationships similar to those found for eurytopic species. The highest number of digger wasp species preferred microhabitats covered with flowering dicotyledonous plants with a small component of woody species (Fig 3b and 3c). Some species, such as *C*. *arenaria*, *C*. *flavilabris*, *N*. *interruptus*, *M*. *bicolor*, *A*. *minor*, *D*. *stigma* are associated with open areas, and forest species *E*. *rubicola* preferred microhabitats where grassland vegetation prevailed (Fig 3b and 3c). The percentage composition of the distinguished species groups in the three analyzed microhabitats is presented in Fig 3a–3c.

### Nesting preferences

The analyzed community of *Sphecidae* was dominated by endogeic (soil-nesting) species. They accounted for 75.43% and 76.00% of the species inventory in the soda ash wastelands in Inowrocław and Janikowo, respectively (Table 2). The percentage of hypergeic (above-ground nesting) species in both cases did not exceed 25%.

### Food preferences

**Fig 4:**
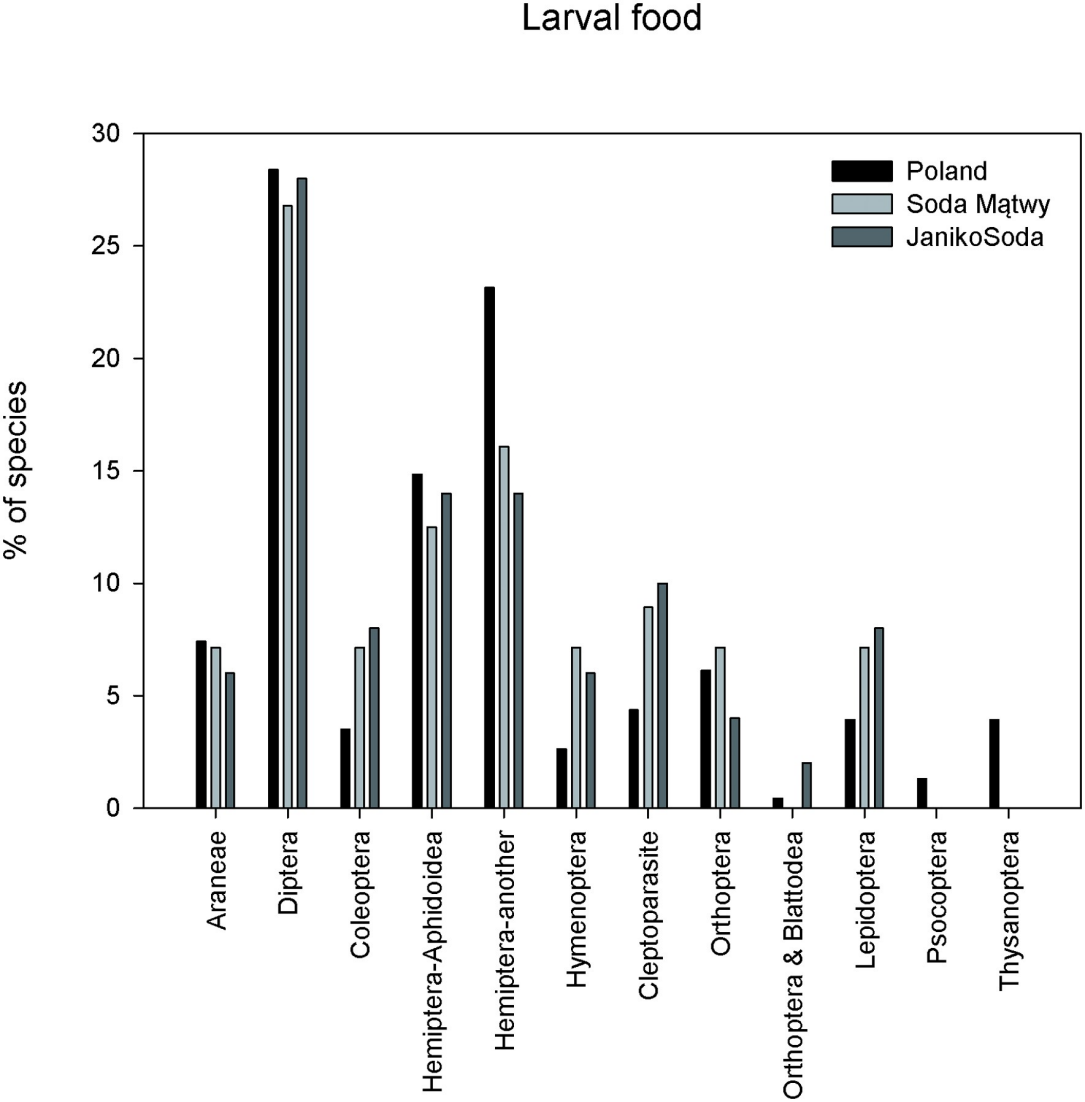
Comparison of the digger wasps larvae food spectrum between soda ash wastelands (Soda Mątwy and JanikoSoda) and whole fauna of polish digger wasps.

Larvae of species of digger wasps we found are characterised by a relatively broad food spectrum including spiders and insects. Most of the wasps (16 species) hunted for flies. Species hunting for flies were also the most common: 987 specimens were trapped (38.39% of all the digger wasps collected). One species hunted *Blattodea*, the only one in Poland. A detailed food spectrum of digger wasp larvae in the soda ash wastelands is presented in Table 2 and Fig 4.

### The changes in the digger wasp associations along the environmental gradients

**Fig 5:**
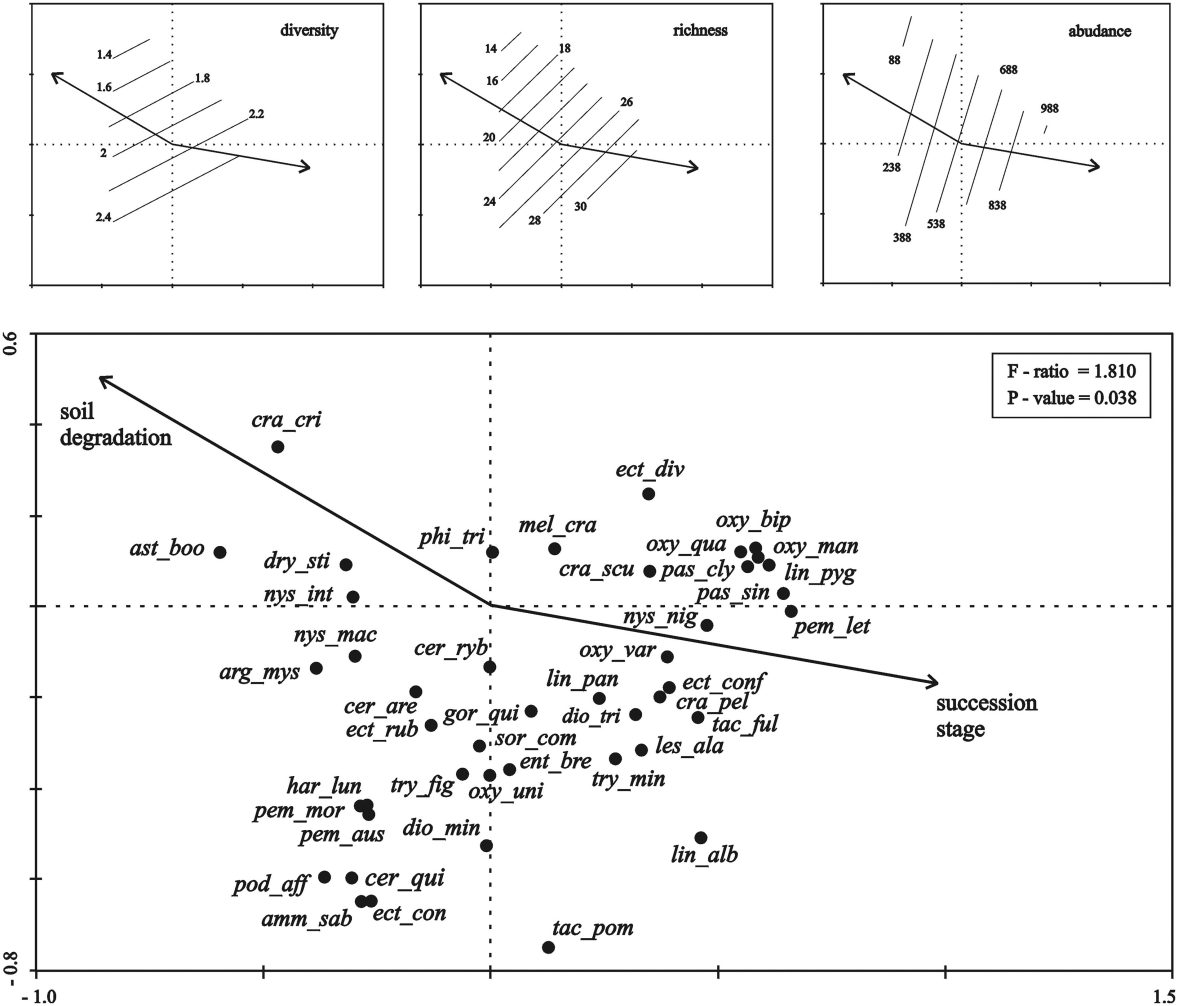
RDA ordination diagram showing the loadings of 44 species on the axes I and II and two "anthropogenic pressure" components (see PCA, Table 1). Changes in the indices describing the structure of digger wasp communities (diversity, species richness and abundance) along the anthropogenic pressure gradient (soil degradation and succession stage) are visualized using a General Linear Model (GLM). Species name abbreviations as in Fig 3.

Digger wasp species differed between sites according to their maturity. As the RDA analysis revealed, the species distribution along the two canonical axes was not random (F-ratio = 1.810, p-value = 0.038, 1,000 permutations). Only 17 out of 64 recorded species were found in microhabitats under the highest pressure (microhabitat 1, of succession stage I, with very moist, salty and alkaline soil; Table 2). The number of recorded species and their abundance increased with the spontaneous succession and the decreasing soil moisture, salinity and alkalinity. Similar relationships are shown for Shannon’s diversity (Fig 5).

## Discussion

Soda ash wastelands, which originated as by-products of industrial processes, provide artificial habitats in the form of anthropogenic islands with unique environmental conditions, constituted by high calcium carbonate content and excessive soil salinity and alkalinity that make these habitats unique in Poland. Our study suggests that postindustrial soda ash dumps can constitute replacement habitats for a diverse and rich digger wasp fauna, and especially for thermophilic species. Psammophilous species accounted for 45.31% of the whole list. The analyzed community of *Sphecidae* was dominated by endogeic (soil-nesting) species (more than 70%; Table 2), whose occurrence is limited by the soil moisture and the availability of open surfaces [47].

The essential role of postindustrial soda ash disposal sites, i.e. habitats under a high anthropogenic pressure, in the protection of digger wasps needs to be recognised because of the high percentage (23.31%) of this species that are rare or endangered (Table 2). At the same time, the high number and abundance of all recorded species points to these sites as valuable replacement habitats for the taxa that prefer open spaces (species associated with open spaces accounted for 73.43% of the inventory; Table 2). Other authors have confirmed the positive role of anthropogenic habitats that support many species [3–4, 7, 12, 18, 31, 48–50].

We demonstrated that the anthropogenic pressure level, defined in terms of soil degradation and the current successional stage, significantly affects the structure of digger wasp communities.

According to our results, fresh, i.e. moist, salty and alkaline dumps as well as the sites of succession stage I are not attractive habitats for most of the digger wasp species.

Excessive soil moisture is an important factor limiting the availability of nesting places suitable for Aculeata [47, 51]. Due to the harsh conditions, such places are dominated by more or less homogenous halophilous vegetation (Table 1, S1 Appendix), that is accompanied by highly specialized but less diversified animal communities [52].

Over time, as spontaneous succession proceeds, the habitat becomes less salty and drier; these changes are correlated with the increase in biodiversity [19, 53].

The reported increase in the species number, abundance and diversity of Spheciformes along the specified environmental gradients was associated both with changes in the physical and chemical soil properties and with spontaneous succession. We have demonstrated that the analyzed and correlated variables: soil salinity, alkalinity and moisture significantly influenced the occurrence of particular species and the structure of Spheciformes. We believe that the soil moisture (a variable correlated with the other ones and therefore removed from the RDA analysis, Table 3) was the main factor limiting the distribution of digger wasps.

The abovementioned relationships are partially consistent with other reports [46, 54–55] showing that dry places with a scarce vegetation cover provide habitats suitable for digger wasps. Microhabitats analyzed in this study that had little plant cover were not attractive as nesting places, most likely because of their excessive soil moisture and salinity.

It seems that the later stages of vegetation development with small woody species is especially favorable for the analyzed fauna, because it promotes not only species associated with open areas, but also stenotopic forest species (Table 2). It is to be expected, however, that with the spontaneous succession toward forest vegetation the diversity of digger wasps would decrease [54]. A similar tendency was observed by Tropek *et al*. [32] in the habitats provided by postindustrial heaps: the number of *Aculeata* decreased with the simplification of the habitat structure due to succession. It has to be kept in mind, however, that the reaction of various invertebrates to the plant succession may vary between habitat types.

We also observed that the habitats of the succession stage I with a small number of flowering plants were hardly penetrated by adult forms of the digger wasps, in spite of an abundant supply of food for the larvae of the analyzed Sphecidae community.

At the same time, a broad spectrum of food for the larvae in the analyzed area (including spiders and insects of eight orders) suggests that postindustrial soda ash basins provide diverse habitats capable of supporting a wide range of animal species (Table 2).

It seems that favorable thermal conditions in soda ash wastelands facilitate the expansion of insects to these areas. In the analyzed habitats we recorded mostly common species widely distributed in Poland. Rare and endangered species comprised only 23.31% of the whole list. The figure is considerably lower than the value of more than 50% reported from the Kampinos National Park Biosphere Reserve [47].

At present, the area of the Park, representing a mosaic of forest and open habitats, provides a refuge for species of the Polish Lowlands [47]. Similarly to the habitats of soda wastes, the area is dominated by thermophilic species nesting in the ground and associated with open habitats [47]. Spheciformes species with similar habitat requirements were also found in the course of studies carried out on spoil tips (coal waste dumps) [3], in sand pits [7], or within the impact zone of motocross sports [56]. The number of species caught in each of these cases was lower than that recorded in the habitats of the soda wastelands. The attractiveness of open habitats of anthropogenic origin was also confirmed for other insect groups. It was shown that, compared to grasslands, railway embankments are richer both in the number and abundance of bee and butterfly species [57]. The sand wasps that dominanted in the habitats of soda wastes are represented by: *Oxybelus quatuordecimnotatus*, *Cerceris rybyensis* and *Crabro cribrarius*. The high relative abundance of these species resulted from favourable habitat conditions. Furthermore, noteworthy is the collection of 17 specimens of *Ectemnius confinis*, which does not belong to species commonly caught in similar habitat types. In Central Europe, the species is classified as highly endangered [23]. A high content of CaCO_3_ (and the lack of stress factors at later succession stages) creates favorable conditions not only for digger wasps but also for other thermophilic insects.

We believe that the analyzed habitats provide replacement habitats for both digger wasps and their potential prey species. Therefore, understanding the mechanisms responsible for the diversity of invertebrates in the analyzed areas is of the utmost importance. The discovered relationships can provide guidelines for the later utilization of basins of soda ash by-products, which are important refuges for fauna species. Furthermore, these results might be the foundation for actions aimed at raising the profile of these unique anthropogenic habitats and to help alleviate the common stereotype of anthropopressure being only a biodiversity-limiting factor. The study also contributed to research in the distribution dynamics of the selected *Aculeata* species in the agricultural environment of the Kujawy region.

## Supporting information

S1 Appendix List of plant species microhabitats occurring within impact of postindustrial soda ash dumping sites and their ecological characteristics. (DOC)

## References

[1] Christie FJ, Hochuli DF. Responses of wasp communities to urbanization: effects on community resilience and species diversity. J. Insect Conserv. 2009;13: 213–221.

[2] Molenda T. The protection of anthropogenic habitats in Poland. J. Ecol. Health. 2013;17(2): 76–80.

[3] Tropek R, Cerna I, Straka J, Cizek O, Konvicka M. Is coal combustion the last chance for vanishing insects of inland drift sand dunes in Europe? Biol. Conserv. 2013;162: 60–64.

[4] Tropek R, Hejda M, Kadlec T, Spitzer L. Local and landscape factors affecting communities of plants and diurnal Lepidoptera in black coal spoil heaps: Implications for restoration management. Ecol. Eng. 2013;57: 252–260.

[5] Twerd L. The industrial areas—as a place rich fauna aculeata. Inż. Ekolog. 2011;27: 219–228.

[6] Babin-Fenske J, Anand M. Patterns of insect communities along a stress gradient following decommissioning of a Cu–Ni smelter. Environ. Pollut. 2011;159: 3036–3043. 10.1016/j.envpol.2011.04.011 21570755

[7] Heneberg P, Bogusch P, Řehounek J. Sandpits provide critical refuge for bees and wasps (Hymenoptera: Apocrita). J. Insect Conserv. 2013;17: 473–490.

[8] Harabiš F, Dolný A. Human altered ecosystems: suitable habitats as well as ecological traps for dragonflies (Odonata): the matter of scale. J. Insect Conserv. 2012;16: 121–130.

[9] Niemelä J, Kotze J, Ashworth A, Brandmayer P, Desender K, New T, et al The search for common anthropogenic impacts on biodiversity: a global network. J. Insect Conserv. 2000;4: 3–9.

[10] Srba M, Heneberg P. Nesting habitat segregation between closely related terricolous sphecid species (Hymenoptera: Spheciformes): key role of soil physical characteristics. J. Insect Conserv. 2012;16: 557–570.

[11] Tropek R, Konvicka M. Can quarries supplement rare xeric habitats in a piedmont region? Spiders of the Blansky les Mts, Czech Republic. Land Degrad. Dev. 2008;19: 104–114.

[12] Tropek R, Kadlec T, Karesova P, Spitzer L, Kocarek P, Malenovsky I, et al Spontaneous succession in limestone quarries as an effective restoration tool for endangered arthropods and plants. J. Appl. Ecol. 2010;47: 139–147.

[13] Niklewska A, Rytelewski J, Brzozowa D. The influence assessment of the chemical plant in Inowroclaw on land located in Notec River valley. Biuletyn Naukowy. 2000;9: 195–203.

[14] Abramski K, Sobolewski J. Ochrona środowiska przed skażeniem ściekami przemysłu sodowego w zbiornikach. Gosp. Wod. 1977;4: 107–110. Polish.

[15] Dyguś KH, Sienkiewicz J. Assessment of vegetation cover on soda waste disposal site at Janikowo, following 13-year-long reclamation. Inż. Ekolog. 2014;36: 65–97.

[16] Siuta J. Effectiveness of reclamation of soda waste disposal site at Janikowo using sewage sludge. Inż. Ekolog. 2014;36: 98–119.

[17] Siuta J, Sienkiewicz R. Rekultywacja terenu składowiska odpadów posodowych w Janikowie. Inż. Ekolog. 2001;3: 43–59. Polish.

[18] Banaszak J, Twerd L. High number of cuckoo wasps (Hymenoptera: Chrysididae) in areas directly affected by lime and sodium industry. Pol. J. Ent. 2010;79: 291–305.

[19] Twerd L. Problems with preservation of biodiversity on the areas transformed by salt and soda industry in the Kujawy region In: Dyguś K, editor. Natural Human Environment–dangers, protection, education; 2012 pp. 275–284.

[20] Potts SG, Vulliamy B, Roberts S, O'Toole C, Dafni A, Ne'eman G, Willmer P. Role of nesting resources in organising diverse bee communities in a Mediterranean landscape. Ecol. Entomol. 2005;30: 78–85.

[21] Cruz-Sánchez M, Asís JD, Gayubo SF, Tormos J, González JA. The effects of wildfire on Spheciformes wasp community structure: the importance of local habitat conditions. J. Insect Conserv. 2011;15: 487–503.

[22] Vieira LC, Oliveira NG, Gayubo SF. On the use of Apiformes and Spheciformes (Insecta: Hymenoptera) populations as a management tool. Biodivers. Conserv. 2011;20(3): 519–530.

[23] Blösch M. Die Grabwespen Deutschlands. Goecke & Evers, Keltern; 2000. German.

[24] Olszewski P, Pawlikowski T. *Passaloecus pictus* Ribaut, 1952 (Hymenoptera: Crabronidae)–first records from Poland. Wiad. Entomol. 2013;32(4): 266–269.

[25] Olszewski P, Pawlikowski T. *Trypoxylon kostylevi* Antropov, 1985 Hymenoptera: Crabronidae) a new species for Poland and the key to Polish species of *Trypoxylon* Latreille, 1796. Pol. J. Ent. 2014;83: 189–199.

[26] Olszewski P, Pawlikowski T, Piekarska-Boniecka H. *Nysson distinguendus* Chevrier, 1867 (Hymenoptera: Crabronidae), a new species to the fauna of Poland. Fragm. Faun. 2013;56(1): 43–46.

[27] Olszewski P, Wiśniowski B, Kostro-Ambroziak A, Pawlikowski T, Piekarska-Boniecka H. *Psenulus meridionalis* Beaumont, 1937, a digger wasp species new to the fauna of Poland (Hymenoptera: Crabronidae). Fragm. Faun. 2013;56(1): 39–42.

[28] Olszewski P, Wiśniowski B, Pawlikowski T, Straka J. *Tachysphex austriacus* Kohl, 1892 (Hymenoptera: Crabronidae) in Poland. Wiad. Entomol. 2013;32(3): 202–206.

[29] Olszewski P, Ljubomirov T, Wiśniowski B, Kowalczyk JK, Krzyżyński M. New records of the genus *Diodontus* Curtis, 1834 (Hymenoptera: Crabronidae), from Bulgaria, Montenegro and Poland, with a key to Central and Eastern European species. Zootaxa. 2016;4061(2): 164–172. 10.11646/zootaxa.4061.2.6 27395490

[30] Wiśniowski B. Annotated checklist of the Polish digger wasps (Hymenoptera: Sphecidae). Pol. J. Ent. 2004;73: 33–63.

[31] Hendrychová M, Šálek M, Červenková A. Invertebrate communities in man-made and spontaneously developed forests on spoil heaps after coal mining. J. Land. Stud. 2008;1: 169–187.

[32] Tropek R, Cerna I, Straka J, Kadlec T, Pech P, Tichanek F, et al Restoration management of fly ash deposits crucially influence their conservation potential for terrestrial arthropods. Ecol. Eng. 2014;73: 45–52.

[33] Szadziewski R. Flies (Diptera) of the saline habitats of Poland. Pol. J. Ent. 1983;53, 31–76.

[34] Ellenberg H, Weber HE, Düll R, Wirth V, Werner W, Paulissen D. Zeigerwerte von Pflanzen in Mitteleuropa. Scr. Geobot. 1992;18: 5–258. German.

[35] Dauber J, Hirsch M, Simmering D, Waldhardt R, Otte A, Wolters V. Landscape structure as an indicator of biodiversity: matrix effects on species richness. Agric. Ecosyst. Environ. 2003;98: 321–329.

[36] Zarzycki K, Trzcińska-Tacik H, Różański W, Szeląg Z, Wołek J, Korzeniak U. Ecological indicator values of vascular plants of Poland. W. Szafer Inst. PAN. Kraków; 2002.

[37] Banaszak J. The review of methods for estimating the numbers of the bees (Hymenoptera: Apoidea). Wiad. Entomol. 1991;10(2): 113–118.

[38] Heneberg P, Bogusch P. To enrich or not to enrich? Are there any benefits of using multiple colors of pan traps when sampling aculeate Hymenoptera? J. Insect Conserv. 2014;18(6): 1123–1136.

[39] Jacobs HJ, Oehlke J. Beiträge zur. Insektenfauna der DDR: Hymenoptera: Sphecidae. 1. Nachtrag. Beitr. Entom. 1990;40: 121–229. German.

[40] Schmid-Egger C, Risch S, Niehuis O. Fauna und Flora in Rheinland Pfalz. Zeitschrifr für Naturschutz, Beiheft 16. Die Wildbienen und Wespen in Rheinland-Pfalz (Hymenoptera, Aculeata). Verbreitung, Ökologie und Gefährdungssituation. Gesellschaft für Naturschutz und Ornithologie, Rheinland-Pfalz e.V. (GNOR), Landau; 1995. German.

[41] Lomholdt O. The Sphecidae (Hymenoptera) of Fennoscandia and Denmark Fauna Entomologica Scandinavica. 4. Second edition E.J. Brill/Scandinavian Science Press, Leiden. Copenhagen; 1984.

[42] Gotelli NJ, Colwell RK. Quantifying biodiversity: procedures and pitfalls in measurment and comparison of species richness. Ecol. Lett. 2001;4: 379–391.

[43] Colwell RK. EstimateS: Statistical estimation of species richness and shared species from sample; 2006. Version 8.0.0. User’s Guide and applications published at: http://purl.oclc.org/estimates

[44] Chao A. Non-parametric estimation of the number of classes in a population. Scand. J. Stat. 1984;11: 265**–**270.

[45] ter Braak CJ, Šmilauer P. CANOCO Reference Manual and User’s Guide to Canoco for Windows: Software for Canonical Community Ordination (version 4). Microcomputer Power (Ithaca, NY, USA); 1998.

[46] Bogdanowicz W, Chudzicka E, Pilipiuk J, Skibińska E, editors. Fauna of Poland. Characteristics and checklist of species. 1. Muzeum i Instytut Zoologii PAN, Warszawa; 2004.

[47] Szczepko K. Ekologia grzebaczowatych (Hymenoptera, Apoidea, Spheciformes) odłogów w Kampinoskim Parku Narodowym. Wyd. Uniw. Łódzkiego, Łódź 2013. Polish.

[48] Benes J, Kepka P, Konvicka M. Limestone quarries as refuges for European xerophilous butterflies. Conserv. Biol. 2003;17: 1058–1069.

[49] Davis BNK. Chalk and limestone quarries as wildlife habitats. Miner. Environ. 1979;1: 48–56.

[50] Vojar J. Colonization of post-mining landscapes by amphibians: A review. Sci. Agric. Bohem. 2006;37: 35–40.

[51] O’Neill KM. Solitary wasps. Behaviour and natural history. Cornell University Press, Ithaca, London; 2001.

[52] Chapman VJ. Salt marshes and salt deserts of the word. London and New York; 1960.

[53] Urbańska J, Urbański K. Seleted aspects of reclamation of soda waste landfill sites. Geomat. Environ. Eng. 2012;6(4): 83–90.

[54] Pawlikowski T, Kruszyński T. Materiały do studiów nad strukturą zespołówżądłówek (Hymenoptera, Aculeata) Polski. Wiad. Entomol. 1996;16(3/4): 165–176. Polish.

[55] Saure C. Bienen und Wespen eines ehemaligen militärischen Übungsgeländes inBerlin-Lichterfelde (Hymenoptera). Märkische Entomologische Nachrichten. 2015;17(1): 1–36. German.

[56] Heneberg P, Bogusch P, Rezáč M. Off-road motorcycle circuits support long-term persistence of bees and wasps (Hymenoptera: Aculeata) of open landscape at newly formed refugia within otherwise afforested temperate landscape. Ecol. Eng. 2016;93: 187–198.

[57] Moroń D, Skórka P, Lenda M, Rożej-Pabijan E, Wantuch M, Kajzer-Bonk J, et al 2014. Railway Embankments as New Habitat for Pollinators in an Agricultural Landscape. PLoS ONE. 2014;9(7): e101297 10.1371/journal.pone.0101297 25054427 PMC4108474

