# Supplementary material for: Can soda ash dumping grounds provide replacement habitats for digger wasps (Hymenoptera, Apoidea, Spheciformes)?
Source: PLoS One. 2017 Apr 19;12(4):e0175664. doi: 10.1371/journal.pone.0175664 (PMC5397032; doi:10.1371/journal.pone.0175664)
Supplement: S1 Appendix — (DOC) [file pone.0175664.s001.doc]

**Appendix 1.** List of plant species microhabitats occurring within impact of postindustrial soda ash dumping sites and their ecological characteristics.

| No | Species | S | **R** | **F** | **microhabitats** | | |
| --- | --- | --- | --- | --- | --- | --- | --- |
|  |  |  |  |  | **1** | **2** | **3** |
| 1 | *Achillea millefolium* L. | 1 | 3-4 | 2-3 | + | + | + |
| 2 | *Agrimonia eupatoria* L. | - | 5 | 2-3 | + | + | + |
| 3 | *Agrostis stolonifera* L. | 1 | 3-5 | 4 | + | + | - |
| 4 | *Anagallis arvensis* L. | - | 3-5 | 3 | + | - | - |
| 5 | *Anchusa officinalis* L. | - | 4 | 3 | - | - | + |
| 6 | *Anthemis arvensis* L. | - | 2-3 | 3 | - | + | - |
| 7 | *Arabis hirsuta* (L.) Scop. | - | 5 | 2 | - | + | - |
| 8 | *Arctium tomentosum* Mill. | - | 4 | 3 | - | - | + |
| 9 | *Artemisia absinthium* L. | - | 3-4 | 2-3 | + | + | + |
| 10 | Artemisia vulgaris L. | - | 4-5 | 3 | + | + | - |
| 11 | *Astragalus* *cicer* L. | - | 5 | 2 | - | - | + |
| 12 | *Atriplex prostrata* Boucher ex DC | - | 4 | 4 | + | - | - |
| 13 | *Berberis vulgaris* L. | - | 5 | 2-3 | - | - | + |
| 14 | *Betula pendula* Roth | - | 3-4 | 3 | - | - | + |
| 15 | *Calamagrostis epigejos* (L.) Roth | - | 3 | 3 | + | + | - |
| 16 | *Calystegia sepium* (L.) R. Br | - | 5 | 4 | + | + | - |
| 17 | *Capsella bursa-pastoris* ([L.](http://pl.wikipedia.org/wiki/Karol_Linneusz)) [Medik.](http://pl.wikipedia.org/wiki/Friedrich_Kasimir_Medikus) | - | 4 | 3 | - | + | - |
| 18 | *Centaurea jacea* L. | 1 | 3-4 | 3 | + | + | + |
| 19 | Cerastium semidecandrum L. | - | 5 | 2 | - | + | - |
| 20 | *Chaerophyllum temulum* L. | - | 4-5 | 4 | + | + | - |
| 21 | *Chelidonium majus* L. | - | 4-5 | 3 | - | - | + |
| 22 | *Cichorium intybus* L. | 1 | 3-5 | 3 | + | - | + |
| 23 | *Cirsium arvense* ([L.](http://pl.wikipedia.org/wiki/Karol_Linneusz)) [Scop.](http://pl.wikipedia.org/wiki/Giovanni_Antonio_Scopoli) | 1 | 3-5 | 2-3 | + | + | + |
| 24 | *Cirsium palustre* ([L.](http://pl.wikipedia.org/wiki/Karol_Linneusz)) [Scop.](http://pl.wikipedia.org/wiki/Giovanni_Antonio_Scopoli) | 1 | 4 | 4 | + | + | + |
| 25 | *Conium maculatum* L. | - | 4 | 3-4 | - | - | + |
| 26 | *Consolida regalis* Gray | - | 5 | 3 | + | - | + |
| 27 | *Conyza canadensis* (L.) [Cronquist](http://pl.wikipedia.org/w/index.php?title=Arthur_John_Cronquist&action=edit&redlink=1) | - | 3-4 | 2-3 | + | - | - |
| 28 | *Cornus sanguinea* L. | - | 4-5 | 2-4 | - | - | + |
| 29 | *Coronilla varia* L. | - | 4-5 | 2 | - | - | + |
| 30 | *Crataegus monogyna* Jacq. | - | 3-5 | 3-4 | - | - | + |
| 31 | *Dactylis glomerata* L. | 1 | 4-5 | 3 | - | + | - |
| 32 | *Daucus carota* L. | 1 | 4-5 | 3 | - | + | + |
| 33 | *Echium vulgare* L. | - | 4-5 | 2 | + | + | + |
| 34 | *Epilobium palustre* L. | 1 | 3 | 5 | + | - | - |
| 35 | *Erodium cicutarium* (L.) L’Hér. | - | 2-3 | 2-3 | - | - | + |
| 36 | *Eryngium planum* L. | - | 5 | 2 | - | - | + |
| 37 | *Eupatorium cannabinum* L. | 1 | 4-5 | 4 | + | + | + |
| 38 | *Euphorbia cyparissias* [L.](http://pl.wikipedia.org/wiki/Karol_Linneusz) | - | 3-5 | 2 | - | - | + |
| 39 | *Euphorbia esula* L. | - | 4-5 | 3 | + | - | - |
| 40 | *Festuca arundinacea* Schreb. | - | 4 | 3-4 | + | + | + |
| 41 | *Festuca ovina* L. | 1 | 3-5 | 2 | - | + | - |
| 42 | *Festuca pratensis* Huds. | 1 | 4 | 3 | + | + | - |
| 43 | *Fumaria officinalis* L. | - | 4 | 3 | - | - | + |
| 44 | *Galium album* Mill. | - | 5 | 2 | + | + | - |
| 45 | *Galium verum* L. | - | 5-4 | 2 | + | + | + |
| 46 | *Geranium molle* L. | - | - | 5 | - | + | - |
| 47 | *Helichrysum arenarium* (L.) Moench | - | 5-3 | 2 | + | + | + |
| 48 | *Hieracium pilosella* L. | - | 2-5 | 2 | - | - | + |
| 49 | *Hordeum murinum* L. | - | 3-4 | 2-3 | - | + | - |
| 50 | *Hypericum perforatum* L. | - | 4 | 2 | - | + | + |
| 51 | *Inula britannica* L. | 1 | 4-5 | 4 | - | - | + |
| 52 | *Knautia arvensis* (L.) J. M. Coult. | - | 5 | 2 | + | - | - |
| 53 | *Lamium album* L. | - | 4 | 3 | - | - | + |
| 54 | *Lamium maculatum* [L.](http://pl.wikipedia.org/wiki/Karol_Linneusz) | - | 4 | 4 | - | - | + |
| 55 | *Lathyrus tuberosus* L. | - | 3-5 | 2-3 | - | - | + |
| 56 | *Lepidium ruderale* L. | 1 | 4 | 2-3 | - | + | - |
| 57 | *Linaria vulgaris* [Mill.](http://pl.wikipedia.org/wiki/Philip_Miller) | - | 3-5 | 2-3 | - | + | - |
| 58 | *Lithospermum arvense* L. | - | 3-4 | 3 | - | + | - |
| 59 | *Lotus corniculatus* L. | 1 | 3-5 | 3-4 | - | - | + |
| 60 | *Matricaria maritima* L. ssp. *inodora* (L.) Dostál | - | 4 | 3 | - | + | - |
| 61 | *Medicago lupulina* L. | - | 3-5 | 2-3 | - | - | + |
| 62 | *Melandrium album* (Mill.) Garcke | 1 | 4 | 3 | - | + | - |
| 63 | *Melilotus alba* Medik. | - | 4 | 2-3 | - | + | + |
| 64 | *Melilotus officinalis* ([L.](http://pl.wikipedia.org/wiki/Karol_Linneusz)) Pall. | - | 4 | 2 | - | + | + |
| 65 | *Myosotis arvensis* (L.) Hill | - | 3-5 | 3 | + | + | - |
| 66 | *Oenothera biennis* L. | - | 3-4 | 2-3 | - | - | + |
| 67 | *Papaver rhoeas* L. | - | 4(5) | 3 | + | + | + |
| 68 | *Plantago intermedia* Gilib. | - | 3-4 | 4 | + | - | - |
| 69 | *Plantago lanceolata* L. | 1 | 4 | 2-4 | - | + | + |
| 70 | *Polygonum lapatifolium* L. subsp. *lapathifolium* | - | 3-4 | 4 | + | - | - |
| 71 | *Potentilla anserina* L. | - | 5-4 | 3-4 | + | + | + |
| 72 | *Potentilla reptans* L. | 1 | 5-4 | 3-4 | + | + | + |
| 73 | *Puccinellia distans* (Jacq.) Parl. | 2 | 4-5 | 4(3) | + | - | - |
| 74 | *Ranunculus arvensis* [L.](http://pl.wikipedia.org/wiki/Karol_Linneusz) | - | 5 | 3 | + | - | - |
| 75 | *Reseda luteola* [L.](http://pl.wikipedia.org/wiki/Karol_Linneusz) | - | 4 | 2-3 | + | + | + |
| 76 | *Rosa canina* [L.](http://pl.wikipedia.org/wiki/Karol_Linneusz) | - | 3-4 | 3-4 | - | + | + |
| 77 | *Salix purpurea* L. | - | 4-5 | 4 | - | - | + |
| 78 | *Sambucus nigra* L. | - | 4 | 3-4 | - | - | + |
| 79 | *Sedum acre* [L.](http://pl.wikipedia.org/wiki/Karol_Linneusz) | - | 3-5 | 1 | - | - | + |
| 80 | *Sedum sexangulare* [L.](http://pl.wikipedia.org/wiki/Karol_Linneusz) | - | 5-4 | 1 | - | - | + |
| 81 | *Senecio vernalis* [Waldst.](http://pl.wikipedia.org/w/index.php?title=Franz_de_Paula_Adam_von_Waldstein&action=edit&redlink=1) & [Kit.](http://pl.wikipedia.org/wiki/Pál_Kitaibel) | - | 3-4 | 2-3 | + | + | + |
| 82 | *Silene nutans* L. | - | 3-5 | 2 | + | - | + |
| 83 | *Sisymbrium loeselii* L. | - | 4-5 | 2 | + | + | + |
| 84 | *Solanum dulcamara* [L.](http://pl.wikipedia.org/wiki/Karol_Linneusz) | 1 | 5-3 | 5-4 | - | + | + |
| 85 | *Solidago gigantea* Aiton | - | - | 3-4 | - | + | + |
| 86 | *Sonchus arvensis* L. | - | (3)4-5 | 3-4 | - | + | + |
| 87 | *Sonchus oleraceus* L. | - | 4 | 3 | + | + | + |
| 88 | *Sorbus aucuparia* L. Emend. Hedl. | - | 2-4 | 3-4 | - | + | - |
| 89 | *Spergularia rubra* (L.) J. Presl & C. Presl | - | 3 | 3-4 | - | - | + |
| 90 | *Tanacetum vulgare* L. | - | 4 | 3-4 | + | + | + |
| 91 | *Taraxacum officinale* F. H. Wigg. | 1 | 4-5 | 3 | + | + | + |
| 92 | *Tragopogon pratensis* L. | - | 4 | 3 | + | - | - |
| 93 | *Trifolium campestre* Schreb. | - | 3-5 | 2 | - | - | + |
| 94 | *Trifolium pratense* L. | 1 | 4 | 3 | + | - | + |
| 95 | *Trifolium repens* [L.](http://pl.wikipedia.org/wiki/Karol_Linneusz) | 1 | 4 | 3-4 | + | - | + |
| 96 | *Triglochin maritimum* [L.](http://pl.wikipedia.org/wiki/L.) | 2 | 5 | 4-5 | + | - | - |
| 97 | *Tussilago farfara* [L.](http://pl.wikipedia.org/wiki/Karol_Linneusz) | - | 4 | 3-4 | + | - | - |
| 98 | *Verbascum thapsus* [L.](http://pl.wikipedia.org/wiki/Karol_Linneusz) | - | 4-5 | 3 | + | + | - |
| 99 | *Veronica chamaedrys* L. | - | 4 | 3 | - | - | + |
| 100 | *Vicia cracca* [L.](http://pl.wikipedia.org/wiki/L.) | - | 4-5 | 3 | - | + | + |
| 101 | *Vicia villosa* Roth | - | 4 | 3 | - | - | + |

The indicator values were averaged for example *Vicia cracca* R : 4-5 = 4.5
